# War Neuroses

**Published:** 1918-04

**Authors:** 


### NERVOUS

---

**War Neuroses.** By John. T. MacCurdy. *Psychiatric Bulletin,* Vol. II, No. 3. July, 1917. Trans. and Abs. by Capt. J. M. Wolfsohn, M. R. C.

MacCurdy defines War Neuroses as " those functional nervous conditions arising in soldiers, which are immediately determined by the conditions of modern warfare and have a symptomatology whose content is directly related to war ".

The term shell-shock, adopted by the British War Office, is not a happy one for two reasons, 1) because it implies a single etiology — the effects of high explosive shells; 2) because it covers too many clinical types.

Twenty-seven cases are described in detail.

MacCurdy finds two main types of neuroses: conditions of anxiety, and those of simple conversion hysteria.

He observes that officers are affected in the proportion of five to one, as compared with privates and N.C.O's.

State of fear may develop, which may last for weeks or months, before the symptoms of the neurosis incapacitate. War neuroses are apparently a corollary of modern methods of fighting.

Individualistic and social or herd instincts, in the psychology of war, are in conflict. In former wars the soldiers suffered great fatigue and exposure, but were compensated by the possibility of gaining satisfaction in hand to hand fighting; this is less common in this war.

MacCurdy feels that the general mechanism of repression of emotionally toned ideas, with their reappearance when repression fails, are responsible for the production of symptoms of war neuroses, and that psycho-analysis is the logical means of treatment. The sex complex is not absent in many of these cases.

The anxiety states, which resemble the neurasthenia in civil practice, are the first signs of the neurosis: fatigue, which is both mental and physical, the former dependent upon the strain of dull routine; constant alertness and speediness of decision; complete self-confidence and spontaneous eagerness, maintained for long periods without sleep, often without food and in the face of constant danger.

The physical influences are represented by the long hours of duty, irregularity of meals, shortness of water, extremes of temperature and exposure.

These constitute what he calls neuro-psychic fatigue, and are added to by certain discomforts, such as presence of vermin and bad odors. Low blood-pressure and temporary symptoms of thyroid disturbance are seen in these cases.

Therefore MacCurdy feels that some chemical change is probably present in these cases of fatigue, which may be the product of some endocrinic reaction to abnormal conditions.

He finds that the more a man's responsibility lies in a mental sphere, the quicker he will be affected, which explains why the anxiety conditions in the pure state, occur especially among officers.

Fatigue is evidenced almost exclusively in the mental sphere, first by a feeling of weariness, then a feeling of tenseness, restless desire for action, irritability, poor concentration. In this state the patient is easily startled. The nocturnal symptoms are: insomnia, hypnagogic hallucinations, dreams, and introspection. Further symptoms in the course of the neurosis are described: fears, the vicious circle resulting, and finally, the idea of escape from the intolerable situation by one of three avenues — an incapacitating wound, being taken prisoner, or being killed. The last is usually the most alluring to these patriotic soldiers; but when the desire for death has become fixed, a complete breakdown is imminent, which occurs after an accident, either mental or physical. Examples are given of this condition.

Concussion in anxiety states is emphasized by " organic " neurologists; but only 1/4 of MacCurdy's cases showed concussion as a preponderant factor, whilst two thirds had no concussion at all in their history.

He has found that a shell dropping among a resting group of men, would produce shell-shock in the one who was awake, whilst those who were asleep showed no bad effects; and repeated concussion when the general condition is perfect, will develop only temporary symptoms, but where fatigue or neurotic symptoms are present, a violent concussion will produce a severe neurosis.

He declares that intelligent treatment is effective in a large number of cases of the anxiety states, but says, " that therapeutic measures must be considered in their psychological effect ".

Conversion hysterias are more common than pure anxiety states and much simpler in mechanism, therefore easier to understand and treat. Conversion hysteria is defined as a neurosis in which there is an alteration, or dissociation of consciousness, regarding some physical function, and is confined almost entirely to privates and N.C.O's. The more important and frequent symptoms, says MacCurdy, are those which provide the patient with relief from active service. Mutism is commonest and is usually followed by aphonia, stammering; motor disturbances are next in order, then deafness; tremors are usually a complication of the anxiety state. Fatigue is the first symptom discoverable, but is not usually so severe as in the anxiety states.

The attitude of antagonism to further fighting, with some idea of release, constitute the backbone of hysteria : concussion, a wound, or physical disease being the *modus operandi* for the development of definite symptoms.

Electricity and massage he considers more often successful than disciplinary measures. Hypnotism has its drawbacks, as it is aimed at the removal of symptoms rather than at the cause.

He discusses heart neuroses, characterized by weakness, dyspnea, palpitation and vertigo; according to some observers this syndrome is possibly a form of war anxiety neurosis.

